# Standardizing Suction Ureteral Access Sheath Technique in Retrograde Intrarenal Surgery (RIRS): Tips, Tricks & Troubleshooting

**DOI:** 10.1590/S1677-5538.IBJU.2025.0535

**Published:** 2025-09-30

**Authors:** Giovanni Scala Marchini, Alexandre Danilovic, Fábio Cesar Miranda Torricelli, Fábio Carvalho Vicentini, Carlos Alfredo Batagello, Rodrigo Perrella, Pedro Antonio Araújo Simões, Alexandre Gilberto Silva, William Carlos Nahas, Eduardo Mazzucchi

**Affiliations:** 1 Faculdade de Medicina da Universidade de São Paulo Hospital das Clínicas Divisão de Urologia São Paulo SP Brasil Seção de Endourologia, Divisão de Urologia, Hospital das Clínicas, Faculdade de Medicina da Universidade de São Paulo, - FMUSP, São Paulo, SP, Brasil

## Abstract

**Introduction:**

Suction ureteral access sheaths (FANS, S-UAS) are reshaping retrograde intrarenal surgery (RIRS) by improving stone-free rates and reducing complications compared to traditional UAS (1–5). Since their use requires significant technical adjustments with limited standardization, we present an instructional video detailing setup, operative choreography, and troubleshooting.

**Methods:**

Single-center instructional case from a tertiary unit. Index patient: 67-year-old man with a 25-mm right pelvic stone (1560 HU; ~3500 mm³). Preoperative considerations included selective prior stenting and off-label α-blockers. We typically use 10/12 or 11/13 Fr suction UAS with 7.5–8.5 Fr flexible ureteroscopes. Setup: pressurized irrigation to the ureteroscope; lateral suction port connected to a labeled collector cup via a vacuum regulator, creating a closed-loop, pressure-aware system. Under fluoroscopy, the sheath is positioned above the ureteropelvic junction (UPJ) with careful advancement into the target calyx. Laser strategy combines dusting and fragmentation with suction. Fragments are evacuated through coordinated suction bursts and slow scope withdrawal. Final inspection defines stent placement and dwell.

**Results:**

Operative time was 115 min, with 25 min of laser use. POD-1 CT confirmed stone-free status. The patient was discharged after 24 h, and the double-J stent with string was removed on day 5. The high-definition video illustrates connections, target pressures, inflow/outflow rules, and provides concise troubleshooting algorithms for common issues: impassable UPJ (use as conventional UAS), friction/kinks, clogging, and system collapse (increase inflow, reduce suction, or reopen outflow).

**Conclusion:**

A standardized suction-UAS technique is feasible and reproducible, optimizing visualization, fragment clearance, pressure control, and safety during RIRS for large stones (6–8). Standardization videos such as this may enhance training, support wider adoption, and improve consistency of outcomes.

**Available at:**VIDEO

## Data Availability

All data generated or analysed during this study are
